# Radiological Followup of the Evolution of Inflammatory Process in Sacroiliac Joint with Magnetic Resonance Imaging: A Case with Pyogenic Sacroiliitis

**DOI:** 10.1155/2012/509136

**Published:** 2012-09-23

**Authors:** Muhammet Cinar, Hatice Tugba Sanal, Sedat Yilmaz, Ismail Simsek, Hakan Erdem, Salih Pay, Ayhan Dinc

**Affiliations:** ^1^Division of Rheumatology, Gulhane Military Medical Academy School of Medicine, 06018 Etlik, Ankara, Turkey; ^2^Department of Radiology, Gulhane Military Medical Academy School of Medicine, 06018 Etlik, Ankara, Turkey

## Abstract

Pyogenic sacroiliitis (PS) is an acute form of sacroiliitis that mostly starts with very painful buttock pain. Here in this case, the followup magnetic resonance (MR) images of a 49-year-old male patient with PS is displayed. After his sacroiliitis was documented by MR images, he was treated with the combination of rifampicin plus streptomycin and moxifloxacin. Serial MR investigations were done to disclose acute and subsequent imaging changes concerning sacroiliac joint and surrounding bone structures. Although after treatment all the symptoms were completely resolved, 20 months later changes suggesting active sacroiliitis on MR images were continuing.

## 1. Introduction

Pyogenic sacroiliitis (PS) is a relatively rare condition, representing only 1-2% of all cases of septic arthritis [1, 2]. Nonspecific initial symptoms and physical examination findings sometimes make it difficult to diagnose, thus delaying appropriate treatment. It is a treatable condition with few long-term complications if appropriate treatment is initiated promptly [3, 4]. Low awareness of the clinical presentation and lack of knowledge of the diagnostic procedure usually lead to delay in diagnosis [2, 4]. Magnetic resonance imaging (MRI) appears to be the most useful imaging technique in evaluating early and subsequent changes involving the joint [5].

It is important to know distinction between infectious and noninfectious sacroiliitis in terms of treatment options. Here, we reported an adult patient who presented with signs, symptoms, and radiologic evidence of unilateral PS that resolved with antibiotic treatment. In this case, we aimed to show the time course of the PS through MR images from the time of first diagnosis to the treatment. In this aspect, this is the first case demonstrating the long-term course of PS with MRI. 

## 2. Case Report

A 49-year-old man without significant medical history was admitted to hospital with a 7-day history of pain in the right hip and buttock, and low-grade fever. He said his pain began acutely one week ago while sleeping. The pain was severe, sharp and progressed over the next several hours. There was no history of trauma. The radiographs showed no abnormalities in the pelvis and hip. The patient was treated with suspected infection in the right hip joint and had been given antibiotics (cefazolin sodium 3 × 500 mg/day plus ciprofloxacin 2 × 500 mg/day) and anti-inflammatory medications on his discharge from the emergency department.

He was admitted to hospital one week after because of worsening buttock pain and associated lower back pain. At the time of admission, he had a mild low-grade fever with a peak temperature of 38°C, and he reported night sweats. The right sacroiliac joint showed tenderness on pelvic compression. Right hip flexion, internal, and external rotation was very painful and limited. The erythrocyte sedimentation rate (ESR), C-reactive protein (CRP), and white blood cell (WBC) count were 65 mm/h, 228 mg/L, and 8.2 × 10^9^/L, respectively. Bone scan showed an increased uptake in the right sacroiliac joint. A Rose Bengal test for brucellosis was positive and a Wright agglutination test titer was >1/320. HLA-B27 was negative. Blood, urine, and stool cultures were negative. 

**Figure 1:**
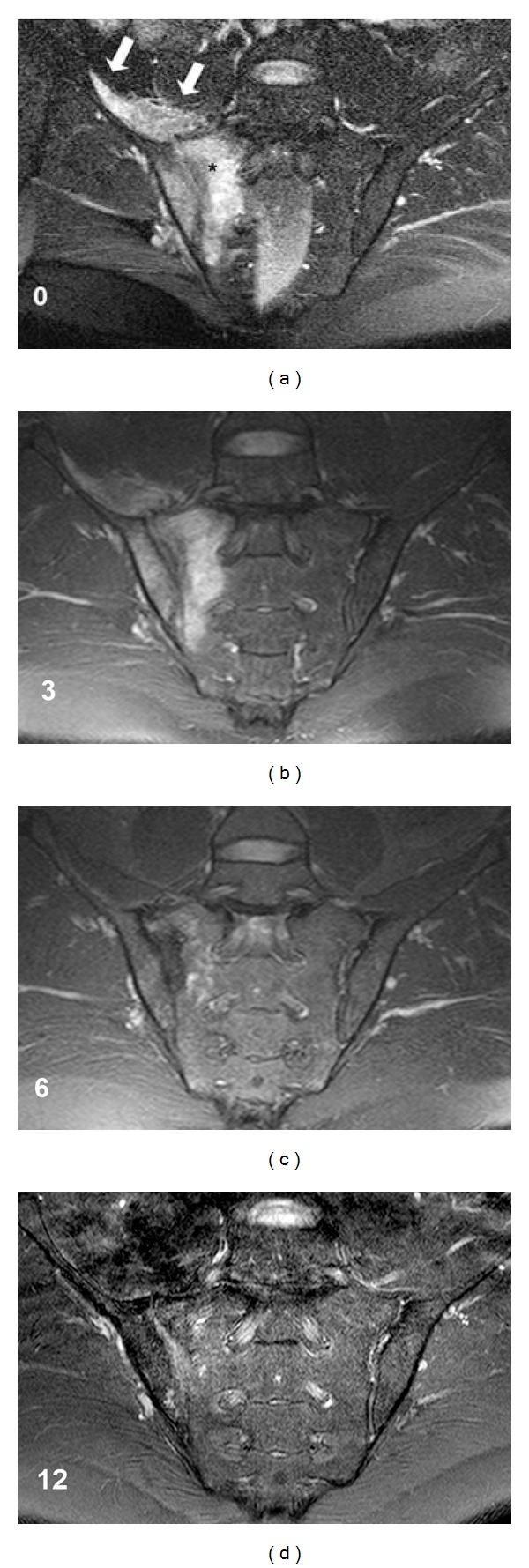
Fluid sensitive sequence on coronal MR images on the first admission (0), and on followups of 3rd, 6th, and 12th months. Bone marrow edema on the right-sided sacroiliac joint is well seen (∗) indicating active inflammation. An accompanying increased signal in the iliopsoas muscle (arrow) is also noticable. Note the diminishing inflammation intensity and distribution in the consecutive months.

**Figure 2:**
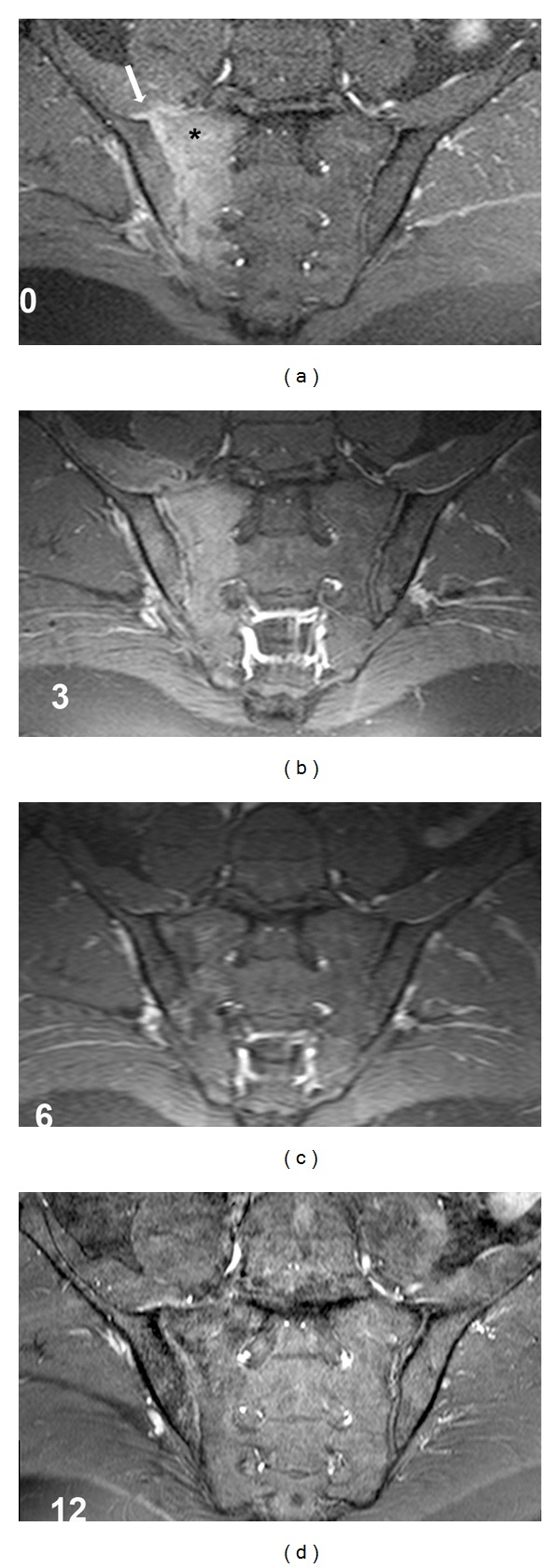
Contrast enhanced images on coronal MR planes through the sacroiliac joints on the first admission (0), and on follow-ups of 3rd, 6th and 12th months. Note the synovitis (arrow) and contrast enhancement indicating active inflammation in the subchondral bone (∗). Contrast enhancement indicating synovitis has been diminished on the 3rd month and disappeared on the consecutive months as well as the bone marrow edema which is more pronounced in the first 3 months.

On MR imaging, mild expansion of the right sacroiliac joint (SIJ) space, along with effusion and bone marrow edema on the joint surfaces indicating active inflammation were detected. An accompanying increased signal in the iliopsoas muscle neighboring right sacroiliac joint was also detected. These findings were yielded to the impression of infective sacroiliitis (Figures 1 and 2). 

The patient was diagnosed as pyogenic sacroiliitis due to brusellosis based on imaging, and clinical and laboratory findings, and he was started on antibiotic therapy with moxifloxacin (400 mg daily, for 2,5 months), rifampicin (600 mg daily for 2.5 months), and streptomycin (1 g daily, for 21 days), along with nonsteroidal anti-inflammatory drugs (NSAIDs). Following treatment his symptoms and complaints improved dramatically. 

Serial MR images were performed to disclose subsequent changes concerning sacroiliac joint and surrounding structures. Three months following the institution of treatment, he had mild pain in the right gluteal region, but no fever and back pain. Acute phase response was within normal limits. At this time on MR images, the signal intensity of inflammation and contrast enhancement pattern at the subchondral bone and the iliopsoas muscle were detected to be mildly decreased. Six months later, he had only mild pain on right gluteal region. After that time, the patient received NSAIDs on as needed basis. Chronic changes (not shown) including joint surface irregularity, sclerosis, and fatty infiltration were first observed on MRI images obtained at this time. Some active inflammatory changes such as bone marrow edema and contrast enhancement were still observed, yet diminished. Same findings were noticeable on MR images on the twelfth month control as well (Figures 1 and 2). Twenty months following the initial diagnosis, bone marrow edema suggesting acute inflammation around the sacroiliac joint was evident on MR images (not shown). These findings were the same with twelfth month control. The patient's clinical and MRI findings and their changes over time were shown in Table 1.

## 3. Discussion

Pyogenic sacroiliitis is considered to be an uncommon condition and can be seen in patients of any age. Female patients tend to have a higher incidence than do male patients, especially in the elderly [2]. The diagnosis of PS requires a degree of clinical suspicion and should be confirmed by imaging methods. It has been reported that the varied presentation of symptoms and signs of PS were always remote from the site of infection [3]. Plain radiograms may be normal in early stages of the disease. In one of the cohort patients with PS, only 63.6% of the patients presented with the full clinical symptoms typical of PS [2]. Therefore, it is suggested that physicians should keep a high index of suspicion while evaluating adult patients with atypical presentations. 

In the previous series, the blood culture yield rate was low. However, local synovial fluid cultures had a high yield rate for pathogens [2]. Synovial fluid aspiration was not universally performed in patients owing to the difficulty in the application of the technique. In our case, we did not perform sacroiliac joint synovial fluid aspiration. Body fluid cultures did not display any bacterial growth. Although the definitive diagnosis requires bacteria isolation from blood or joint fluid cultures, acute onset, one-sided involvement of the joints, and severe gluteal pain accompanied by high fever as in this case were considered as findings supporting PS. Therefore, MR imaging is probably the imaging method of choice to detect PS [6–8]. It provides a detailed evaluation of the joint and surrounding soft tissues [3, 8]. As in our case, together with clinical findings, unilateral involvement, bone marrow edema adjacent to the SIJ surfaces and edema in the neighborhood soft tissues help to diagnose and distinguish infectious from noninfectious sacroiliitis (i.e., some spondyloarthropathies). After a course of intravenous antibiotics, the symptoms completely resolved, thus supporting our diagnosis of infective sacroiliitis. 

MR imaging of SIJs has evolved as the most relevant imaging modality for diagnosing early SIJ changes, particularly in patients with ankylosing spondylitis (AS). Active inflammatory lesions such as bone marrow edema (BME)/osteitis, synovitis, enthesitis, and capsulitis can be detected by MRI. Among these, the clear presence of BME/osteitis was considered essential for defining active sacroiliitis. Structural damage lesions such as sclerosis, erosions, fat deposition, and ankylosis can also be detected by MRI, which are accepted as chronic changes [9]. Although the course of MRI changes was comprehensively defined for patients having AS, little is known for patients with infective sacroiliitis. 

In this case, consecutive MRIs of a 49-year-old male diagnosed with PS was displayed. We showed that although clinical findings disappear within three months, the MRI findings of suggesting active sacroiliitis did not resolve completely. Even twenty months following the initial diagnosis, bone marrow edema suggesting acute inflammation around the sacroiliac joint was evident on MR images. Interestingly, the patient did not have any complaints at this time (Table 1). We did not perform histologic evaluation to correlate the imaging and clinical findings. Indeed, there is a lack in the literature concerning infective sacoiliitis and its clinical and histologic findings in the time course. Histology-based studies to match the MR imaging findings were conducted mainly in patients with noninfective sacroiliitis [10, 11]. In one of these studies, despite the findings of positive histopathologic results indicating active inflammation, interestingly, it was reported that no MRI findings may be observed [10]. 

In conclusion, our case demonstrated that in patients with infective sacroiliitis, correlation of the clinical and radiological findings might be far from being perfect. Considering this case, it is obvious that radiological recovery takes longer and it would be more appropriate to rely on clinical and laboratory findings instead of radiological images for the followup of those patients.

## Figures and Tables

**Table 1:** Clinical course of pyogenic sacroiliitis.

	0 Mo	3 Mo	6 Mo	12 Mo	20 Mo
Clinic findings					
(i) Fever	+	−	−	−	−
(ii) Night sweat	+	−	−	−	−
(iii) Right gluteal pain	Very severe	Moderate	Mild	No pain	No pain
(iv) Pain with sacroiliac stretch	Very severe	Mild	No pain	No pain	No pain
(v) Limitation of spinal mobility measurements	−	−	−	−	−
WBC, ×10^9^/L	8.2	5.1	7.5	6.1	6.6
ESR, mm/h	65	4	5	5	5
CRP, mg/L	228	3,32	2.66	<3.4	<3.4
Rose Bengal	Positive	Positive	Negative	Not studied	Not studied
Wright agglutination	>1/320	1/160	<1/80	Not studied	Not studied
MRI findings					
(i) Expansion of the sacroiliac joint space	++	++	+	−	−
(ii) Joint effusion of posteroinferior part of the joint	+	±	−	−	−
(iii) Bone marrow edema on the iliac and sacral surfaces	++++	+++	++	+	+
(iv) Sclerosis and irregularity	−	−	+	+	+
(v) Fatty bone marrow changes	−	−	+	+++	+++
(vi) Edematous changes on neighborhood muscles	+	+	−	−	−
Treatment					
(i) NSAID use	Used	Used	On demand	On demand	Not used
(ii) Antibiotics	Used	Not used	Not used	Not used	Not used

The plus (+) and minus (−) signs were used instead of absent and present meaning. The severity of MRI findings are shown with plus sign. ++++ is defined as very high severity; +++ as high severity; ++ as moderate severity; and + as mild severity.
